# Multivariate Analyses to Assess the Effects of Surgeon and Hospital Volume on Cancer Survival Rates: A Nationwide Population-Based Study in Taiwan

**DOI:** 10.1371/journal.pone.0040590

**Published:** 2012-07-17

**Authors:** Chun-Ming Chang, Kuang-Yung Huang, Ta-Wen Hsu, Yu-Chieh Su, Wei-Zhen Yang, Ting-Chang Chen, Pesus Chou, Ching-Chih Lee

**Affiliations:** 1 Department of Surgery, Buddhist Dalin Tzu Chi General Hospital, Chiayi, Taiwan; 2 School of Medicine, Tzu Chi University, Hualian, Taiwan; 3 Division of Allergy, Immunology, and Rheumatology, Department of Internal Medicine, Buddhist Dalin Tzu Chi General Hospital, Chiayi, Taiwan; 4 Division of Hematology-Oncology, Department of Internal Medicine, Buddhist Dalin Tzu Chi General Hospital, Chiayi, Taiwan; 5 Cancer center, Buddhist Dalin Tzu Chi General Hospital, Chiayi, Taiwan; 6 Department of Medical Research, Buddhist Dalin Tzu Chi General Hospital, Chiayi, Taiwan; 7 Division of Metabolism and Endocrinology, Department of Internal Medicine, Buddhist Dalin Tzu Chi General Hospital, Chiayi, Taiwan; 8 Community Medicine Research Center and Institute of Public Health, National Yang-Ming University, Taipei, Taiwan; 9 Department of Otolaryngology, Buddhist Dalin Tzu Chi General Hospital, Chiayi, Taiwan; 10 Center for Clinical Epidemiology and Biostatistics, Buddhist Dalin Tzu Chi General Hospital, Chiayi, Taiwan; Virginia Commonwealth University School of Medicine, United States of America

## Abstract

**Background:**

Positive results between caseloads and outcomes have been validated in several procedures and cancer treatments. However, there is limited information available on the combined effects of surgeon and hospital caseloads. We used nationwide population-based data to explore the association between surgeon and hospital caseloads and survival rates for major cancers.

**Methodology:**

A total of 11677 patients with incident cancer diagnosed in 2002 were identified from the Taiwan National Health Insurance Research Database. Survival analysis, the Cox proportional hazards model, and propensity scores were used to assess the relationship between 5-year survival rates and different caseload combinations.

**Results:**

Based on the Cox proportional hazard model, cancer patients treated by low-volume surgeons in low-volume hospitals had poorer survival rates, and hazard ratios ranged from 1.3 in head and neck cancer to 1.8 in lung cancer after adjusting for patients’ demographic variables, co-morbidities, and treatment modality. When analyzed using the propensity scores, the adjusted 5-year survival rates were poorer for patients treated by low-volume surgeons in low-volume hospitals, compared to those treated by high-volume surgeons in high-volume hospitals (*P*<0.005).

**Conclusions:**

After adjusting for differences in the case mix, cancer patients treated by low-volume surgeons in low-volume hospitals had poorer 5-year survival rates. Payers may implement quality care improvement in low-volume surgeons.

## Introduction

Cancer is a leading cause of death worldwide and it accounted for 7.6 million deaths (13% of all deaths) in 2008 [1]. In Western countries as well as Taiwan, lung cancer, breast cancer, colorectal cancer, prostate cancer, and head and neck cancer are the most common causes of malignant tumors [1], [2], [3]. Cancer treatment is now a serious socioeconomic problem and an important public health issue which deserves more attention.

A positive association with caseload volume and outcomes has been observed in many procedures and cancer surgeries [4], [5], [6]. Previous studies have indicated that increased numbers of procedures for hospitals or surgeons were associated decreased perioperative morbidity and complications, or shortened length of stay [4], [7]. For lung cancer, breast cancer, and colon cancer surgeries, patients who underwent treatment at hospitals or with surgeons that perform a large number of procedures are likely to survive longer than others [8], [9], [10] [11], [12]. Part of this phenomenon could be explained by the understanding that “practice makes perfect;” “selective referral” may be an alternative explanation in other cases [6], [13]. Most studies explored the benefits of increased caseload and cancer operative mortality or survival rates at the hospital level or surgeon level. The combined effects of surgeon and hospital caseloads on cancer operative mortality rates has been explored in the past, but there is little data available on the combined effects of surgeon and hospital caseloads on cancer survival rates [14], [15].

The purpose of this study was to test the hypothesis that cancer patients treated by low-volume surgeons in low-volume hospitals incur poor survival rates, compared with those treated by high-volume surgeons in high-volume hospitals.

## Materials and Methods

### Ethics Statements

This study was initiated after being approved by the Institutional Review Board of Buddhist Dalin Tzu Chi General Hospital, Taiwan. Because the identification numbers and personal information of the individuals included in the study were not included in the secondary files, the review board stated that written consent from patients was not required.

### Patients and Study Design

**Figure 1:**
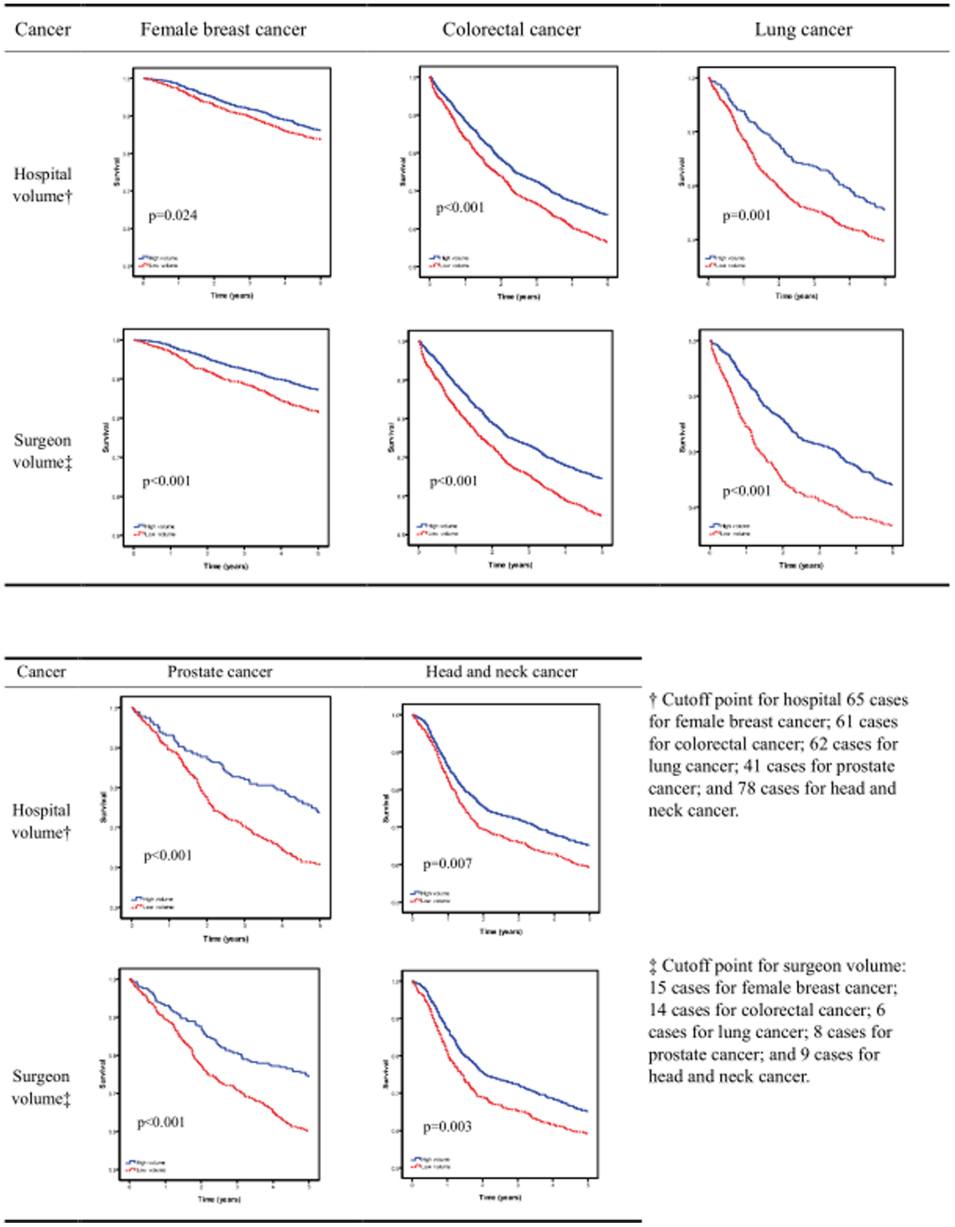
Procedure for definition of hospital volume and surgeon volume. (1) Hospitals were further categorized by their total patient volume by using unique hospital identifiers in this database. The cancer patients fell in to three approximately equal groups based on hospital volume. (2) Surgeons were further categorized by their total patient volume by using unique hospital identifiers in this database. The cancer patients fell in to three approximately equal groups based on surgeon volume. (3) These cancer patients were then linked to the death data extracted from the catastrophic illness and cancer dataset covering the years 2002–206. (4) 5-year survival of the cancer patients were analyzed according to hospital volume or surgeon volume stratified by tumor sites. (5) In Lung cancer and prostate cancer, survival curves of the medium and low hospital/surgeon group were similar. So the medium and low groups were merged as the “low volume”. (6) In breast cancer, colorectal cancer, and head and neck cancer, survival curves of the high and medium hospital/surgeon group were similar. So the high and medium groups were merged as the “high volume”. (7) The cutoff point of each cancer in hospital level or surgeon level was derived from the above procedures (5) and (6).

We used data from 2002 to 2006 from the National Health Insurance (NHI) Research Database, which covered medical benefit claims for over 23 million people in Taiwan (approximately 97 percent of the island’s population). Patients with major cancers in Taiwan, including breast cancer, colorectal cancer, lung cancer, prostate cancer, and head and neck cancer, who received surgical treatment with or without adjuvant therapy in the year 2002 were included (Appendix S1). Physicians were sorted by their total patient volumes using unique physician identifiers in this database. Patients were assigned to be treated by low-volume and high-volume surgeons. Hospitals were sorted using similar methods Figure 1) [16]. Detailed procedures for defining high or low caseload were explained in Figure 1. The cancer patient data was then linked to mortality data for the years 2002 to 2006.

### Measurements

The key dependent variable of interest was the 5-year survival rate of cancer patients. The key independent variables were the combined effects of surgeon and hospital caseloads, which were sorted into groups based on volume (high volume hospital-high volume surgeon, high volume hospital-low volume surgeon, low volume hospital-high volume surgeon, and low volume hospital-low volume surgeon). Patient demographics included age, gender, geographic location, treatment modality, severity of disease, and individual socioeconomic status. The comorbidities of each patient was based on the modified Charlson Comorbidity Index score, which has been widely used in recent years for risk adjustment in administrative claims data sets [17].

This study used enrollee category (EC) as a proxy measure of socioeconomic status (SES), which is an important prognostic factor for cancer [18], [19]. The cancer patients were classified into 3 subgroups: high SES (civil servants, full-time or regular paid personnel with a government affiliation or employees of privately owned institutions), moderate SES (self-employed individuals, other employees, and members of the farmers’ or fishermen’s associations), and low SES (veterans, low-income families, and substitute service draftees) [20].

### Statistical Analysis

The SAS statistical package (version 9.2; SAS Institute, Inc., Cary, N.C.) and SPSS (version 15, SPSS Inc., Chicago, IL, USA) were used to analyze data. A p-value of *P*<0.05 was used to determine statistical significance.

The cumulative 5-year survival rates and the survival curves were constructed and compared using a log-rank test. Survival was measured from the time of cancer diagnosis by using overall death as censoring variables. The Cox proportional regression model and the survival analysis with propensity score stratification were used to compare outcomes between different physician caseloads.

### (1) Cox Proportional Hazards Model

The Cox proportional regression model was used to evaluate the combined effect of surgeon and hospital volume on survival rates after adjusting for demographic variables and treatment modalities. The goodness of fit of the regression model was evaluated by the deviance of −2 Log Likelihood.

### (2) Propensity Score

Propensity score stratification was applied to replace the wide host of confounding factors that may be present in an observational study with a variable of these factors [21], [22], [23], [24]. To derive the propensity score in this study, patient characteristics were entered into a logistic regression model predicting selection for different category of the providers. The characteristics included the year in which the patient was diagnosed, age, gender, the Charlson Comorbidity Index score, geographic area of residence, and treatment modality. The effect of caseload assignment on the 5-year survival rate was analyzed within each quintile. The Mantel-Haenszel odds ratio was calculated in addition performing the Cochran-Mantel-Haenszel χ^2^ test.

## Results

A total of 3620 deaths (31%) were identified from the total sample of 11677 patients that underwent curative surgery with or without adjuvant therapy between 2002 and 2006. The characteristics of the patients are summarized in Table 1. 5933 (50.8%) cancer patients were treated by high-volume surgeons in high-volume hospitals, 1392 (11.9%) by low-volume surgeons in high-volume hospitals, 1591(13.6%) in high-volume surgeons in low-volume hospitals and 2761 (22.9%) in low-volume surgeons in low-volume hospitals. Patients treated by low-volume surgeons in low-volume hospitals were more likely to be older, reside in suburban and rural areas, live in central, southern and eastern Taiwan, and to have lower socioeconomic statuses. Table 2 showed the association of surgeons and hospitals. High-volume surgeons were more likely to perform surgeries in high-volume hospitals among breast cancer, colorectal cancer, prostate cancer and head and neck cancer treatment.

**Table 1:** Baseline characteristics according to hospital volume and surgeon volume (n = 11677).

Variable	High volume hospital†		Low volume hospital†		*p* value*
	High volume surgeon‡ (n = 5933)	Low volumesurgeon‡ (n = 1392)	High volume surgeon‡ (n = 1591)	Low volumesurgeon‡ (n = 2761)	
Mean age, years (±SD)	57±14.1	58±14.5	59±13.8	62±14.6	<0.001
Gender					<0.001
Female (%)	3425 (57.7)	756 (54.3)	768 (48.3)	1308 (47.4)	
Male (%)	2508 (42.3)	636 (45.7)	823 (51.7)	1453 (52.6)	
Urbanization					<0.001
Urban (%)	1983 (33.4)	466 (33.5)	486 (30.5)	675 (24.4)	
Suburban (%)	2562 (43.2)	588 (42.2)	728 (45.8)	1285 (46.5)	
Rural (%)	1388 (23.4)	338 (24.3)	377 (23.7)	801 (29.0)	
Geographic region					<0.001
Northern	2835 (47.8)	675 (48.5)	766 (48.1)	1187 (43.0)	
Central	1643 (27.7)	326 (23.4)	436 (27.4)	747 (27.1)	
Southern and Eastern	1455 (24.5)	391 (28.1)	389 (24.5)	827 (30.0)	
Socioeconomic status					<0.001
High	2969 (50.0)	658 (47.3)	761 (47.8)	1164 (42.2)	
Medium	2396 (40.4)	563 (40.4)	660 (41.5)	1220 (44.2)	
Low	568 (9.6)	171 (12.3)	170 (10.7)	377 (13.7)	
Charlson Comorbidity Index Score					
0 (%)	3223 (54.3)	753 (54.1)	959 (60.3)	1723 (62.4)	<0.001
1–6 (%)	2131 (35.9)	494 (35.5)	498 (31.3)	834 (30.2)	
>6 (%)	579 (9.8)	145 (10.4)	134 (8.4)	204 (7.4)	
Adjuvant therapy					0.035
Nil (%)	3461 (58.3)	799 (57.4)	936 (58.8)	1634 (59.2)	
Radiotherapy (%)	525 (8.8)	120 (8.6)	207 (13.0)	363 (13.1)	
Chemotherapy (%)	1104 (18.6)	286 (20.5)	236 (14.8)	426 (15.4)	
Chemoradiotherapy (%)	843 (14.2)	187 (13.4)	212 (13.3)	338 (12.2)	

* One way ANOVA test for continuous variable (mean age); Pearson’s chi-square test for categorical variables.

† Cutoff point for hospital 65 cases for female breast cancer; 61 cases for colorectal cancer; 62 cases for lung cancer; 41 cases for prostate cancer; and 78 cases for head and neck cancer.

‡ Cutoff point for surgeon volume: 15 cases for female breast cancer; 14 cases for colorectal cancer; 6 cases for lung cancer; 8 cases for prostate cancer; and 9 cases for head and neck cancer.

**Table 2:** The distribution of surgeons and hospitals.

	High volume surgeon		Low volume surgeon		*p* value
	n	%	n	%	
**Female Breast Cancer**					<0.001
High volume hospital	46	73	106	30	
Low volume hospital	17	27	248	70	
**Colorectal Cancer**					<0.001
High volume hospital	65	77	137	34	
Low volume hospital	19	23	266	66	
**Lung Cancer**					0.738
High volume hospital	7	25	20	22	
Low volume hospital	21	75	71	78	
**Prostate Cancer**					<0.001
High volume hospital	13	62	34	14	
Low volume hospital	8	38	212	86	
**Head and Neck Cancer**					<0.001
High volume hospital	32	68	102	36	
Low volume hospital	15	32	184	64	

### Cox Proportional Hazards Model Analysis

**Figure 2:**
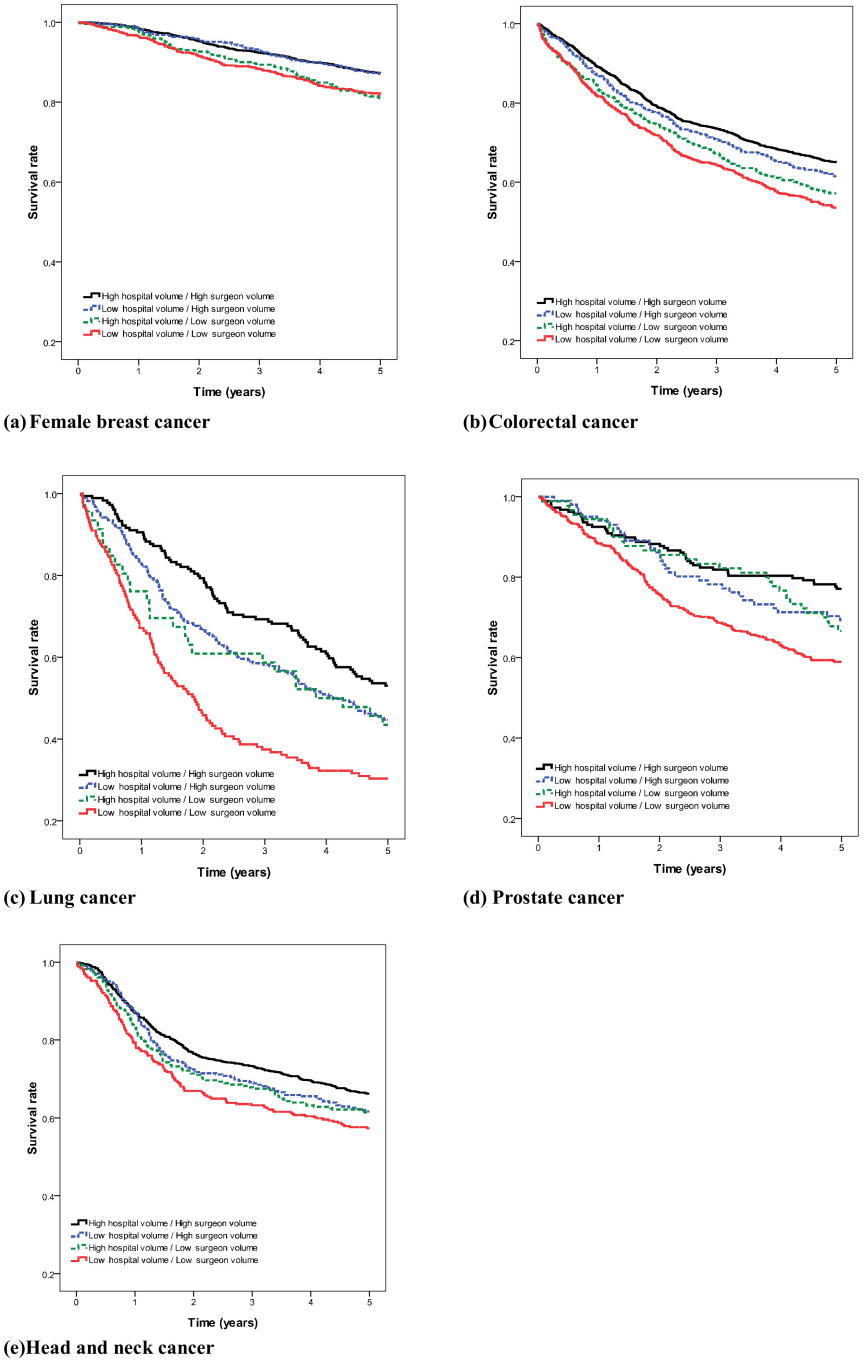
Cancer survival rates by combined effect of surgeon and hospital caseloads (a) Breast cancer (b) Colorectal cancer (c) Lung cancer (d) Prostate cancer (e) Head and neck cancer.

Table 3 shows the combined effects of surgeon and hospital caseloads on 5-year survival rates. Patients treated by low volume surgeons in low-volume hospitals had the poorest survival rates (Figure 2a–e). Table 4 shows the adjusted hazard ratios based on the Cox proportional hazards regression model after adjusting for patient comorbidities, geographic location, type of residence, and treatment modalities. The negative association between survival and surgeon and hospital caseloads remained statistically significant in the multivariate analysis. To ensure the observed effect of volume is not influenced by older age and comorbidites, we repeat the Cox regression analysis after sequentially removing age and comorbidites. Model A (without age and Charlson Comorbidity Index Score) in Table 5 showed the impact of provider volume remained robust. Cancer patients treated by low-volume surgeons and low-volume hospitals had poorer survival rates for major cancers.

**Table 3:** Five-year survival rate according to hospital volume and surgeon volume (n = 11677).

Variable	High volume hospital†		Low volume hospital†		*p* value*
	High volume surgeon‡ (n = 5933)	Low volumesurgeon‡ (n = 1392)	High volume surgeon‡ (n = 1591)	Low volumesurgeon‡ (n = 2761)	
Female breast cancer (%)	87.3	81.0	87.1	82.0	<0.001
Colorectal cancer (%)	65.1	57.2	61.5	53.5	<0.001
Lung cancer (%)	53.1	43.5	44.7	30.3	<0.001
Prostate cancer (%)	77.1	66.7	69.3	58.9	<0.001
Head and neck cancer (%)	66.3	61.4	61.6	57.3	0.006

* Log-rank test for 5-year survival rates.

† Cutoff point for hospital 65 cases for female breast cancer; 61 cases for colorectal cancer; 62 cases for lung cancer; 41 cases for prostate cancer; and 78 cases for head and neck cancer.

‡ Cutoff point for surgeon volume: 15 cases for female breast cancer; 14 cases for colorectal cancer; 6 cases for lung cancer; 8 cases for prostate cancer; and 9 cases for head and neck cancer.

**Table 4:** Cox regression according to hospital volume and surgeon volume (n = 11677).

Surgeon volume‡	Provider Caseload							
	Hospital volume ≧ cutoff† (n = 7325)				Hospital volume < cutoff† (n = 4352)			
	No. of deaths/No. of cases	Adjusted hazardratio**	95% CI	*p* value*	No. of deaths/No. of cases	Adjusted Hazard ratio**	95% CI	*p* value*
**Female breast cancer**								
High volume surgeon	282/2213	1.00			53/411	1.24	0.92–1.67	0.155
Low volume surgeon	93/490	1.44	1.14–1.83	0.002	154/843	1.67	1.37–2.05	<0.001
**Colorectal cancer**								
High volume surgeon	829/2372	1.00			192/499	1.22	1.04–1.43	0.012
Low volume surgeon	208/486	1.34	1.15–1.56	<0.001	426/917	1.53	1.36–1.72	<0.001
**Lung cancer**								
High volume surgeon	84/179	1.00			152/275	1.10	0.83–1.46	0.517
Low volume surgeon	26/46	1.33	0.85–2.08	0.208	108/155	1.82	1.35–2.46	<0.001
**Prostate cancer**								
High volume surgeon	43/188	1.00			31/101	1.31	0.82–2.09	0.264
Low volume surgeon	30/90	1.19	0.74–1.92	0.478	202/492	1.77	1.25–2.49	0.001
**Head and neck cancer**								
High volume surgeon	331/981	1.00			117/305	1.19	0.96–1.47	0.117
Low volume surgeon	108/280	1.06	0.85–1.32	0.633	151/354	1.30	1.07–1.58	0.009

* *p* value for adjusted hazard ratios in Cox regression model.

† Cutoff point for hospital 65 cases for female breast cancer; 61 cases for colorectal cancer; 62 cases for lung cancer; 41 cases for prostate cancer; and 78 cases for head and neck cancer.

‡ Cutoff point for surgeon volume: 15 cases for female breast cancer; 14 cases for colorectal cancer; 6 cases for lung cancer; 8 cases for prostate cancer; and 9 cases for head and neck cancer.

** Adjusted for patients’ age, gender, socioeconomic status, urbanization and region of residence, Charlson Comorbidity Index Score, and adjuvant therapy.

**Table 5:** The adjusted hazard ratios of provider category in different regression model.

	Null model	Model A†		Model B‡			
		HR	95%CI	HR			95%CI
**Female Breast Cancer**							
High volume surgeon/High volume hospital		1		1			
High volume surgeon/Low volume hospital		1.09	0.81–1.47	1.24		0.92–1.67	
Low volume surgeon/High volume hospital		1.58	1.25–2.00	1.44		1.14–1.83	
Low volume surgeon/Low volume hospital		1.60	1.31–1.95	1.67		1.37–2.05	
*−2Log Likelihood*	9552	9403		9204			
**Colorectal Cancer**							
High volume surgeon/High volume hospital		1		1			
High volume surgeon/Low volume hospital		1.15	0.98–1.35	1.22	1.04–1.43		
Low volume surgeon/High volume hospital		1.31	1.12–1.52	1.34	1.15–1.56		
Low volume surgeon/Low volume hospital		1.37	1.22–1.54	1.53	1.36–1.72		
−*2Log Likelihood*	26929	26723		26122			
**Lung Cancer**							
High volume surgeon/High volume hospital		1		1			
High volume surgeon/Low volume hospital		1.03	0.78–1.37	1.10	0.83–1.46		
Low volume surgeon/High volume hospital		1.36	0.87–2.13	1.33	0.85–2.08		
Low volume surgeon/Low volume hospital		1.80	1.34–2.43	1.82	1.35–2.46		
−*2Log Likelihood*	4534	4425		4375			
**Prostate Cancer**							
High volume surgeon/High volume hospital		1		1			
High volume surgeon/Low volume hospital		1.34	0.84–2.13	1.31	0.82–2.09		
Low volume surgeon/High volume hospital		1.32	0.83–2.11	1.19	0.74–1.92		
Low volume surgeon/Low volume hospital		1.78	1.27–2.49	1.77	1.25–2.49		
−*2Log Likelihood*	4021	3959		3815			
**Head and Neck Cancer**							
High volume surgeon/High volume hospital		1		1			
High volume surgeon/Low volume hospital		1.17	0.94–1.45	1.19	0.96–1.47		
Low volume surgeon/High volume hospital		1.05	0.84–1.31	1.06	0.85–1.32		
Low volume surgeon/Low volume hospital		1.25	1.03–1.52	1.30	1.07–1.58		
*−2Log Likelihood*	10391	10134		10089			

Abbreviation: HR, hazard ratio; 95% CI, 95% confidence interval.

† Adjusted for patients’ gender, socioeconomic status, urbanization and region of residence, and adjuvant therapy.

‡ Adjusted for patients’ age, Charlson Comorbidity Index Score, gender, socioeconomic status, urbanization and region of residence, and adjuvant therapy.

### Propensity Score Analysis

Stratification according to propensity scores and assessment of the combined effects of surgeon and hospital caseloads on survival were performed among the patients treated by high-volume surgeons in high-volume hospitals and low-volume surgeons in low-volume hospitals (Appendix S2). Table 6 shows the survival rates for both caseload groups after stratification. In most situations, patients treated by high-volume surgeons in high-volume hospitals had higher 5-year survival rates. The p-value for Cochran-Mantel-Haenszel statistics comparing survival rates for low-volume surgeons in low-volume hospitals and high-volume surgeons in high-volume hospitals, controlling for propensity scores, was <0.001. The patients treated by low-volume surgeons in low-volume hospitals had higher mortality rates. The adjusted 5-year survival rates for patients treated by low-volume surgeons in low-volume hospitals were lower than patients treated by high-volume surgeons in high-volume hospitals.

**Table 6:** Adjusted five-year survival rate stratified by tumor.

Cancer		Provider Caseload		*p* value*	Mantel-Haenszel adjusted odds ratio (95% CI)
		High volume surgeon/High volume hospital(%)	Low volume surgeon/Low volume hospital (%)		
Female breast cancer	87.5		81.1	<0.001	1.65 (1.32–2.06)
Colorectal cancer	65.1		53.8	<0.001	1.64 (1.40–1.92)
Lung cancer	50.2		39.5	<0.001	1.67 (1.02–2.73)
Prostate cancer	67.1		60.8	<0.001	1.62 (1.07–2.46)
Head and neck cancer	66.1		58.4	0.001	1.41 (1.10–1.81)

* *p* value for Mantel-Haenszel chi-square test.

In summary, cancer patients treated by low-volume surgeons in low-volume hospitals had poorer survival rates. The result was robust as the survival rates were determined using both the Cox proportional regression model and stratification by propensity scores.

## Discussion

Patients who underwent treatment by low-volume physicians in low-volume hospitals had lower survival rates. The adjusted hazard ratio ranged from 1.3 in head and neck cancer to 1.8 in lung cancer. This negative association remained statistically significant when analyzed using propensity scores. Payers should conduct some interventions and sponsor quality improvement research.

Benefits have been associated with increased caseloads in the treatment of acute myocardial infarction, transphenoid surgery, shoulder surgery, carotid endarterectomy, etc [25], [26]. The magnitude of the volume-outcome association varied greatly in different procedures [15]. Previous studies have explored the positive association of high physician or hospital volume on cancer survival rates or perioperative mortality and length of stay [4], [7], [8], [9], [27], [28]. Several studies from Taiwan have reported the positive association of surgeon caseload or hospital caseload and cancer outcomes [16], [29], [30]. However, only a few studies have reported on the combined effects of physician and hospital caseloads on cancer survival [14]. Our study reported the results of the combined effects of surgeon and hospital caseloads, and the results were validated using two different multivariate analyses.

The quality of the risk-adjustment technique in analyzing administrative information is an important issue [31]. The Cox proportional hazard model was used to evaluate the effect of different combinations of physician and hospital caseloads. Cancer patients treated by low-volume physicians in low-volume hospitals were found to have a higher risk of mortality after adjusting for comorbidities, and other confounding factors. However, there were some differences with regard to age, and clinical condition between different caseload groups. In the second part of our series propensity scores were used to stratify the patients into five groups with similar propensity scores in order to reduce the effects of selection bias between the different caseload groups [22], [23], [32]. Cancer patients treated by low-volume physicians in low-volume hospitals were found to have poorer outcomes. Differences in the case mix and caring process between high- and low-volume providers may explain some of results we observed [12].

In Taiwan, and most other countries, cancer treatment is conducted by a team. In fact, high-volume physicians represent high-volume teams. It is possible that high-volume physicians, who coordinate large experienced teams, including radiation oncologists, hematology oncologists, radiologists, and specialized nurses, are of paramount importance for the treatment of cancer. Several hypotheses for the volume-outcome relationship have been proposed. The “practice makes perfect” concept suggests that increased caseloads may help physicians or hospital staff develop skills and execute treatment procedures more effectively, as is the case with surgical procedures, chemotherapy, irradiation, and manipulation of radiation oncology teletherapy units. Achieving complete excision with a tumor-free margin with regional lymph node dissection is crucial in the treatment of most cancers. A positive surgical margin and regional lymph node metastasis are the most significant predictive factors for breast cancer, lung cancer, colorectal cancer, and oral cancer [33], [34], [35], [36], [37]. Adequate and well-performed regional lymph node dissection and a successful complete excision of the primary tumor may be the crucial procedures for success in cancer treatment. High-volume surgeons may have the surgical skills and experience necessary to perform these procedures. In treating early-stage breast cancer, Gilligan et al. [38]. reported that high-volume surgeons were significantly more likely to provide care which adheres to National Institutes of Health recommendations because of the higher volume of axillary lymph node dissection patients who underwent either breast-conserving surgery or mastectomy. High-volume surgeons are more likely to cooperate with fixed hematology oncologists who are familiar with chemotherapy, determining cycles of chemotherapy, choice of chemotherapy regimen, and treatment of complications. The radiation oncologists on a high-volume team may also be more familiar with appropriate radiation doses [24].

Adherence to treatment guidelines could be one of the reasons why high-volume providers have better outcomes. High-volume physicians may use effective treatment strategies more often than low-volume physicians [25]. High-volume surgeons also often adopted multi-disciplinary approaches, while low-volume surgeons were less likely to interact with oncologists or attend multi-disciplinary meetings for breast cancer series [39]. Combined therapy utilization may also be one of the reasons for better outcomes in high-volume physicians who treated cancer. Low-volume physicians in low-volume hospitals may not follow international treatment guidelines. For cancer treatment, the combined effects of low-volume surgeons and low-volume hospitals reached the highest hazard ratio of 1.8 in lung cancer. Resection of lung cancer and the subsequent intensive care is the corner stone of lung cancer surgery treatment. Lung cancer treatment relies on surgeon experience, hospital hardware and well-trained staff members, which emphasizes the effects and importance of both surgeon and hospital volume [8].

Socioeconomic status may affect the patient’s choice of hospitals or surgeons through several mechanisms [40], [41]. Cancer patients with low SES were more likely to seek medical advice or undergo treatment in regional or district hospitals, and low-caseload hospitals, which were negative prognostic factors in cancer survival rates [40]. This could be due to the unequal distribution of hospital resources. Patients with low SES were more likely to reside in suburban or rural areas in which there were fewer medical centers or large public hospitals. Furthermore, cancer patients with low SES were less likely to choose high-volume providers due to a lack of health care information.

The “selective referral hypothesis” suggests that high-volume physicians may be chosen by healthier patients or patients with early-stage diseases [6]. It is also possible that high-volume provider are referred sicker patients or patients with advanced stage, which would actually strengthen the results of our study [42]. This is true in this dataset. High-volume provider treated cancer patients with higher Charlson Comorbidity Index Score (Table 1).

How could our findings be applied to policy intervention? First, it may be beneficial to limit the performance of cancer surgery to medical centers or high-volume providers. This approach had been endorsed by a number of researchers [11], [43], [44]. Second, research organizations and payers, such as the Bureau of National Health Insurance, may sponsor clinical quality improvement research to identify care and treatment strategy differences between providers with different caseloads. Treatment strategies of high-volume surgeons in high-volume hospitals may be analyzed and put into practice in other areas around the country in order to improve survival rates. Third, for high-volume physicians, payers may encourage them, or consider using incentive measures, to serve as expert consultants to low-volume physicians in low-volume hospitals in order to improve healthcare quality and survival rates. Fourth, public interventions, such as treatment guidelines or quality of care reports for hospitals could be offered to cancer patients, especially for those in low SES or in suburban/rural areas. Fifth, quality improvement in cancer care, such as multidisciplinary conferences, implementation of institutional governance procedures, and standardization of cancer-care, could be conducted in low-volume hospitals [45], [46]. However, we have to know that encouraging payer to reward or punish hospitals and surgeons is a double edge sword. One third to one half cancer patients in this study were treated by low-volume surgeons or hospitals. Shifting cancer resection from low to high provider may destabilize low and rural hospitals and surgeons.

Our study has several limitations. First, the relationship of the stages of the different cancers and provider caseloads could not be assessed because cancer stage data was not included in the database. However, Begg et al. revealed that cancer stage and patient age were independent of caseload volume in a SEER-Medicare linked database [5]. Second, using surgeon volume as a surrogate may have some limitation. The appearance of a low volume surgeon may be attributed to that some surgeons operate in more than one hospital. Among the National Health Insurance system in Taiwan, this phenomenon is rare. Third, the observed variation may be attributed to coding errors or code creeping, and the information on postoperative complications, length of hospital stay and re-admission rates may be added in the further studies [47]. Fourth, instead of cancer-specific survival rates, the overall survival rate was used. But, Roohan et al. reported no significant difference between survival models for overall survival and breast cancer-specific survival rates [10]. Fifth, the extreme high volume provider may have negative effect. However, we used dichotomized volume for analysis which prevented us to answer this question. Another limitation of our study is the issue of over-fitting when we established cutoffs by the previous methods [16]. A better method such as taking a random sample of 25–50% of the cancer patients in the database and apply this methodology used to determine the cutoffs for each cancer for low and high volumes, and then validate the methodology and cutoffs by examining the remaining cancer patients with those cutoffs. Given the robust magnitude of the effects and statistical significance of the effects in this study; however, these limitations are unlikely to compromise our results.

In summary, our findings provide support for the combined effects of surgeon and hospital caseload volume with regard to survival outcomes for major cancers. After analysis via the Cox proportional hazard model and propensity scores, there was a clear association between low-volume surgeons in low-volume hospitals and poorer 5-year survival rates. Treatment strategies adopted by high-volume physicians may be further analyzed and utilized to improve overall survival rates of cancer patients. Payers may encourage low-volume physicians to participate in more training workshops and follow cancer treatment guidelines in order to improve patients’ survival rates.

## Supporting Information

Appendix S1 **Operation code included in this study.**
(DOC)

Appendix S2 **Five-year survival rate in different propensity score strata.**
(DOC)

## References

[1] WHO media centre (2008). Accessed 2011 January 2.. http://www.who.int/mediacentre/factsheets/fs297/en/index.html.

[2] Jemal A, Siegel R, Ward E, Hao Y, Xu J (2009). Cancer Statistics, 2009.. CA: A Cancer Journal for Clinicians.

[3] Bureau of Health Promotion, Department of Health, ROC (2011). Cancer statistics in Taiwan.. http://www.bhp.doh.gov.tw/BHPnet/Portal/Statistics.

[4] Gruen RL, Pitt V, Green S, Parkhill A, Campbell D (2009). The Effect of Provider Case Volume on Cancer Mortality: Systematic Review and Meta-Analysis.. CA: A Cancer Journal for Clinicians.

[5] Begg C, Cramer L, Hoskins W, Brennan M (1998). Impact of hospital volume on operative mortality for major cancer surgery.. JAMA.

[6] Harold S Luft, Sandra S Hunt, Maerki SC (1987). The volume-outcome relationship: practice-makes-perfect or selective-referral patterns?. Health Services Research.

[7] Goodney P, Stukel T, Lucas F, Finlayson E, Birkmeyer J (2003). Hospital volume, length of stay, and readmission rates in high-risk surgery.. Ann Surg.

[8] Bach PB, Cramer LD, Schrag D, Downey RJ, Gelfand SE (2001). The Influence of Hospital Volume on Survival after Resection for Lung Cancer.. New England Journal of Medicine.

[9] Schrag D, Cramer LD, Bach PB, Cohen AM, Warren JL (2000). Influence of Hospital Procedure Volume on Outcomes Following Surgery for Colon Cancer.. JAMA: The Journal of the American Medical Association.

[10] Roohan PJ, Bickell NA, Baptiste MS, Therriault GD, Ferrara EP (1998). Hospital volume differences and five-year survival from breast cancer.. Am J Public Health.

[11] Luft HS, Bunker JP, Enthoven AC (1979). Should operations be regionalized? The empirical relation between surgical volume and mortality.. N Engl J Med.

[12] Halm E, Lee C, Chassin M (2002). Is volume related to outcome in health care? A systematic review and methodologic critique of the literature.. Ann Intern Med.

[13] Cheng SH, Song HY (2004). Physician performance information and consumer choice: a survey of subjects with the freedom to choose between doctors.. Qual Saf Health Care.

[14] Schrag D, Earle C, Xu F, Panageas KS, Yabroff KR (2006). Associations Between Hospital and Surgeon Procedure Volumes and Patient Outcomes After Ovarian Cancer Resection.. Journal of the National Cancer Institute.

[15] Birkmeyer JD, Stukel TA, Siewers AE, Goodney PP, Wennberg DE (2003). Surgeon Volume and Operative Mortality in the United States.. New England Journal of Medicine.

[16] Lin CC, Lin HC (2008). Effects of surgeon and hospital volume on 5-year survival rates following oral cancer resections: the experience of an Asian country.. Surgery.

[17] Deyo RA, Cherkin DC, Ciol MA (1992). Adapting a clinical comorbidity index for use with ICD-9-CM administrative databases.. Journal of Clinical Epidemiology.

[18] Braaten T, Weiderpass E, Lund E (2009). Socioeconomic differences in cancer survival: the Norwegian Women and Cancer Study.. BMC Public Health.

[19] Kwok J, Langevin SM, Argiris A, Grandis JR, Gooding WE (2010). The impact of health insurance status on the survival of patients with head and neck cancer.. Cancer.

[20] Chen CY, Liu CY, Su WC, Huang SL, Lin KM (2007). Factors Associated With the Diagnosis of Neurodevelopmental Disorders: A Population-Based Longitudinal Study.. Pediatrics.

[21] Joffe MM, Rosenbaum PR (1999). Invited Commentary: Propensity Scores.. Am J Epidemiol.

[22] Rubin DB (1993). Tasks in statistical inference for studying variation in medicine.. Med Care.

[23] Rubin DB (1997). Estimating causal effects from large data sets using propensity scores.. Ann Intern Med.

[24] Lee CC, Huang TT, Lee MS, Su YC, Chou P (2011). Survival rate in nasopharyngeal carcinoma improved by high caseload volume: a nationwide population-based study in Taiwan.. Radiation Oncology.

[25] Thiemann D, Coresh J, Oetgen W, Powe N (1999). The association between hospital volume and survival after acute myocardial infarction in elderly patients.. N Engl J Med.

[26] Barker FG, Klibanski A, Swearingen B (2003). Transsphenoidal Surgery for Pituitary Tumors in the United States, 1996–2000: Mortality, Morbidity, and the Effects of Hospital and Surgeon Volume.. Journal of Clinical Endocrinology & Metabolism.

[27] Kee F, Shields R, Wilson RH, Harper C, Patterson CC (1999). Influence of hospital and clinician workload on survival from colorectal cancer: cohort studyCommentary: How experienced should a colorectal surgeon be?. BMJ.

[28] Renzulli P, Lowy A, Maibach R, Egeli RA, Metzger U (2006). The influence of the surgeon’s and the hospital’s caseload on survival and local recurrence after colorectal cancer surgery.. Surgery.

[29] Chen CS, Liu TC, Lin HC, Lien YC (2008). Does high surgeon and hospital surgical volume raise the five-year survival rate for breast cancer? A population-based study.. Breast Cancer Res Treat.

[30] Lien YC, Huang MT, Lin HC (2007). Association between surgeon and hospital volume and in-hospital fatalities after lung cancer resections: the experience of an Asian country.. Ann Thorac Surg.

[31] Davoli M, Amato L, Minozzi S, Bargagli AM, Vecchi S (2005). [Volume and health outcomes: an overview of systematic reviews].. Epidemiol Prev.

[32] D’Agostino RB (1998). Propensity score methods for bias reduction in the comparison of a treatment to a non-randomized control group.. Stat Med.

[33] Soerjomataram I, Louwman MW, Ribot JG, Roukema JA, Coebergh JW (2008). An overview of prognostic factors for long-term survivors of breast cancer.. Breast Cancer Res Treat.

[34] Hyndman ME, Mullins JK, Pavlovich CP (2010). Pelvic node dissection in prostate cancer: extended, limited, or not at all?. Current Opinion in Urology 20: 211–217 210.1097/MOU.1090b1013e328338405d.

[35] Tai P, Tonita J, Yu E, Skarsgard D (2003). Twenty-year follow-up study of long-term survival of limited-stage small-cell lung cancer and overview of prognostic and treatment factors.. International Journal of Radiation Oncology*Biology*Physics.

[36] Woolgar JA (2006). Histopathological prognosticators in oral and oropharyngeal squamous cell carcinoma.. Oral Oncology.

[37] Larsen SR, Johansen J, Soensen JA, Krogdahl A (2009). The prognostic significance of histological features in oral squamous cell carcinoma.. J Oral Pathol Med.

[38] Gilligan MA, Neuner J, Sparapani R, Laud PW, Nattinger AB (2007). Surgeon Characteristics and Variations in Treatment for Early-Stage Breast Cancer.. Arch Surg.

[39] Stefoski Mikeljevic J, Haward RA, Johnston C, Sainsbury R, Forman D (2003). Surgeon workload and survival from breast cancer.. Br J Cancer.

[40] Lee CC, Chien SH, Hung SK, Yang WZ, Su YC (2012). Effect of individual and neighborhood socioeconomic status on oral cancer survival.. Oral Oncol.

[41] Taylor CB, Ahn D, Winkleby MA (2006). Neighborhood and Individual Socioeconomic Determinants of Hospitalization.. American journal of preventive medicine.

[42] Al-Refaie WB, Muluneh B, Zhong W, Parsons HM, Tuttle TM (2012). Who Receives Their Complex Cancer Surgery at Low-Volume Hospitals?. Journal of the American College of Surgeons.

[43] Grumbach K, Anderson GM, Luft HS, Roos LL, Brook R (1995). Regionalization of cardiac surgery in the United States and Canada. Geographic access, choice, and outcomes.. JAMA.

[44] Hillner BE, Smith TJ, Desch CE (2000). Hospital and physician volume or specialization and outcomes in cancer treatment: importance in quality of cancer care.. J Clin Oncol.

[45] Becher EC, Chassin MR (2002). Taking Health Care Back: The Physician’s Role in Quality Improvement.. Academic Medicine.

[46] Scott IA, Poole PJ, Jayathissa S (2008). Improving quality and safety of hospital care: a reappraisal and an agenda for clinically relevant reform.. Internal Medicine Journal.

[47] Virnig BA, McBean M (2001). Administrative data for public health surveillance and planning.. Annual Review of Public Health.

