# Supplementary material for: Multivariate Analyses to Assess the Effects of Surgeon and Hospital Volume on Cancer Survival Rates: A Nationwide Population-Based Study in Taiwan
Source: PLoS One. 2012 Jul 17;7(7):e40590. doi: 10.1371/journal.pone.0040590 (PMC3398946; doi:10.1371/journal.pone.0040590)
Supplement: Appendix S1 — Operation code included in this study. (DOC) [file pone.0040590.s001.doc]

**Appendix S1** Operation code included in this study

1. Female breast cancer:

Excision or destruction of breast tissue, not otherwise specified: 85.20 – 85.25,

*Reduction mammoplasty and subcutaneous mammectomy: 85.30,

Other unilateral subcutaneous mammectomy, Bilateral subcutaneous mammectomy

with synchronous implant, Other bilateral subcutaneous mammectomy: 85.34 –

85.36, *Mastectomy, Unilateral simple mastectomy, Bilateral simple

mastectomy, Unilateral extended simple mastectomy, Bilateral extended simple

mastectomy, Unilateral radical mastectomy, Bilateral radical mastectomy, 85.40 –

Unilateral extended radical mastectomy, Bilateral extended radical mastectomy:

85.48

2. Colorectal cancer:

Endoscopic excision or destruction of lesion of duodenum, Other local excision of lesion of duodenum, Other destruction of lesion of duodenum, Local excision of lesion or tissue of small intestine, except duodenum, Other destruction of lesion of small intestine, except duodenum: 45.30 – 45.34,

*Local excision or destruction of lesion or tissue of large intestine, Excision of lesion or tissue of large intestine: 45.40 – 45.41,

Endoscopic destruction of other lesion or tissue of large intestine: 45.43,

Other destruction of lesion of large intestine: 45.49,

*Other excision of small intestine, Multiple segmental resection of small intestine, Other partial resection of small intestine, Total removal of small intestine: 45.60 – 45.63,

*Partial excision of large intestine, Multiple segmental resection of large intestine: 45.70 – 45.71,

Right hemicolectomy, Resection of transverse colon, Left hemicolectomy, Sigmoidectomy: 45.73 – 45.76,

Other partial excision of large intestine, Total intra-abdominal colectomy: 45.79 – 45.80,

Resection of exteriorized segment of large intestine: 46.04,

*Local excision or destruction of lesion or tissue of rectum: 48.30,

Destruction of rectal lesion or tissue by laser, Destruction of rectal lesion or tissue by cryosurgery, Local excision of rectal lesion or tissue: 48.33 – 48.35,

Soave submucosal resection of rectum: 48.41,

Abdominoperineal resection of rectum: 48.50,

*Other resection of rectum, Transsacral rectosigmoidectomy: 48.60 – 48.61,

Other anterior resection of rectum, Posterior resection of rectum, Duhamel resection of rectum: 48.63 – 48.65,

Other resection of rectum: 48.69,

*Incision or excision of perirectal tissue or lesion, Incision of perirectal tissue, Excision of perirectal tissue: 48.80 – 48.82

3. Lung cancer:

*Local excision or destructionof lesion or tissue of bronchus, Endoscopic excision

or destruction of lesion or tissue of bronchus: 32.00 – 32.01,

*Local excision or destruction of lesion or tissue of lung: 32.2,

Endoscopic excision or destruction of lesion or tissue of lung, Other local excision

or destruction of lesion or tissue of lung, Segmental resection of lung: 32.28 –

32.30,

Lobectomy of lung: 32.4,

Complete pneumonectomy: 32.5,

Other excision of lung: 32.9

4. Prostate cancer:

*Transurethral prostatectomy, Transurethral (ultrasound) guided laser induced

prostatectomy(TULIP): 60.20 – 60.21,

Other transurethral prostatectomy, Suprapubic prostatectomy: 60.29 – 60.30,

Retropubic prostatectomy: 60.4,

Radical prostatectomy: 60.5,

*Other prostatectomy, Local excision of lesion of prostate, Perineal

prostatectomy: 60.60 – 60.62,

Other prostatectomy: 60.69,

*Incision or excision of periprostatic tissue: 60.80,

Vasectomy: 63.73

5. Head and neck cancer:

Excision or destruction of lesion of nose, not otherwise specified, Local excision or

destruction of intranasal lesion, Local excision or destruction of other lesion of

nose: 21.30 – 21.32,

*Frontal sinusotomy and sinusectomy: 22.4

Excision or destruction of lesion or tissue of tongue, Partial glossectomy, Complete

glossectomy, Radical glossectomy: 25.1 – 25.4

*Excision of lesion or tissue of bony palate, Local excision or destruction of lesion

or tissue of bony palate, Wide excision or destruction of lesion or tissue of bony

palate, *Excision of other parts of mouth: 27.3 – 27.4,

Other excision of mouth: 27.49,

Radical neck dissection, not otherwise specified, Radical neck dissection, unilateral,

Radical neck dissection, bilateral: 40.40 – 40.42,

Radical excision of lymph nodes, not otherwise specified, Radical excision of

axillary lymph nodes, Radical excision of periaortic lymph nodes, Radical excision

of iliac lymph nodes, Radical groin dissection: 40.50 – 40.54,

Radical excision of other lymph nodes: 40.59

Pharyngeal diverticulectomy, Pharyngectomy(partial): 29.32 – 29.33,

Other excision or destruction of lesion or tissue of pharynx: 29.39

*Excision or destruction of lesion or tissue of larynx: 30.0,

Other excision or destructionof lesion or tissue of larynx, Hemilaryngectomy,

*Other partial laryngectomy, Epiglottidectomy, Vocal cordectomy: 30.09 – 30.22,

Other partial laryngectomy, Complete laryngectomy: 30.29 – 30.3,

Radical laryngectomy: 30.4
