# Supplementary material for: Multivariate Analyses to Assess the Effects of Surgeon and Hospital Volume on Cancer Survival Rates: A Nationwide Population-Based Study in Taiwan
Source: PLoS One. 2012 Jul 17;7(7):e40590. doi: 10.1371/journal.pone.0040590 (PMC3398946; doi:10.1371/journal.pone.0040590)
Supplement: Appendix S2 — Five-year survival rate in different propensity score strata. (DOC) [file pone.0040590.s002.doc]

| **Appendix S2**. Five-year survival rate in different propensity score strata | | | | | |
| --- | --- | --- | --- | --- | --- |
| Propensity score stratum | | Provider caseload | | P value | Mantel-Haenszel adjusted  odds ratio  (95% CI) |
|  |  | High volume hospital / High volume surgeon  (%) | Low volume hospital / Low volume surgeon  (%) |  |  |
| Female breast cancer | | |  |  | 1.65 (1.32 – 2.06) |
|  | I | 83.7 | 80.2 | 0.324 |  |
|  | II | 87.5 | 76.1 | 0.001 |  |
|  | III | 87.4 | 82.3 | 0.092 |  |
|  | IV | 91.0 | 84.1 | 0.015 |  |
|  | V | 88.1 | 82.7 | 0.060 |  |
| Colorectal cancer | | |  |  | 1.64 (1.40 – 1.92) |
|  | I | 55.2 | 51.2 | 0.272 |  |
|  | II | 70.5 | 59.7 | 0.007 |  |
|  | III | 69.9 | 56.6 | 0.001 |  |
|  | IV | 66.0 | 49.5 | <0.001 |  |
|  | V | 63.9 | 52.2 | 0.002 |  |
| Lung cancer | |  |  |  | 1.67 (1.02 – 2.73) |
|  | I | 66.7 | 63.6 | 0.687 |  |
|  | II | 45.5 | 65.0 | 0.260 |  |
|  | III | 25.0 | 17.5 | 0.073 |  |
|  | IV | 66.7 | 35.3 | 0.006 |  |
|  | V | 47.1 | 16.0 | 0.012 |  |
| Prostate cancer | |  |  |  | 1.62 (1.07 – 2.46) |
| Grade | I | 91.0 | 76.3 | 0.021 |  |
|  | II | 83.3 | 73.6 | 0.200 |  |
|  | III | 82.8 | 64.2 | 0.056 |  |
|  | IV | 37.5 | 46.8 | 0.516 |  |
|  | V | 40.9 | 43.0 | 0.847 |  |
| Head and neck cancer | | |  |  | 1.41 (1.10 – 1.81) |
|  | I | 68.9 | 70.4 | 0.939 |  |
|  | II | 68.3 | 60.3 | 0.119 |  |
|  | III | 69.1 | 46.2 | <0.001 |  |
|  | IV | 63.8 | 60.0 | 0.527 |  |
|  | V | 60.3 | 55.3 | 0.452 |  |
|  | | | | | |
